# Correlation between Preoperative Anxiety and ABO Blood Types: Evidence from a Clinical Cross-Sectional Study

**DOI:** 10.1155/2019/1761693

**Published:** 2019-12-02

**Authors:** Feng Xu, Jiang-wen Yin, Er-feng Xiong, Hong He, Qing-tong Zhang, Shi-wen Fan, Xin-lei Qin, Sheng Wang

**Affiliations:** ^1^Department of Anesthesiology, First Affiliated Hospital, School of Medicine, Shihezi University, Shihezi, China; ^2^Department of Anesthesiology, People's Hospital of Xinjiang Uygur Autonomous Region, Xinjiang, China; ^3^Department of Anesthesiology, First Affiliated Hospital of USTC, Division of Life Sciences and Medicine, University of Science and Technology of China, Hefei, Anhui 230001, China

## Abstract

Gene-environment interaction is identified as the determinant in anxiety. ABO blood types represent a part of the genetic phenotype. Therefore, we assume ABO blood types correlate with preoperative anxiety. This cross-sectional study enrolled 352 patients with different ABO blood types, scheduled for elective surgery between 2018 and 2019 in the First Affiliated Hospital of Shihezi University. HADS (hospital anxiety and depression scale) scores and VA (visual analogue scales for anxiety) scores were all used to assess the preoperative anxiety in the A, B, AB, and O groups. Bivariate correlation and logistic regression were performed to identify relationships between preoperative anxiety and related variables. A significant difference in VA and HADS-A (anxiety) scores was found between the AB and other groups. The ratio of preoperative anxiety was 3.73 (95% CI [confidence interval]: 2.32-6.00, *P* < 0.001) times in female than in male; 0.36 (95% CI: 0.21-0.63, *P* < 0.001) times in ASA (American Society of Anesthesiologists) grade II than in grade I; 0.41 (95% CI: 0.20-0.86, *P* < 0.05) times in ASA grade III than in grade I; 1.25 (95% CI: 1.1-1.41, *P* < 0.001) times in higher VAS (visual analogue scales for pain) scores than in lower VAS scores; and 0.28 (95% CI: 0.16-0.49, *P* < 0.01) times in non-AB blood type than in AB blood type. Differences in ABO blood types were found in preoperative anxiety, and the AB group displayed a high preoperative anxiety level. ABO blood types, sex, ASA grade, and VAS were associated with preoperative anxiety. This trial is registered with ChiCTR1800019390.

## 1. Introduction

During the preoperative period, when surgical procedure and anesthetic were determined, preoperative anxiety is induced by fear and worry of anesthesia and operation, pain, death, family and prognosis [1–3]. The current study reported that preoperative anxiety, with high prevalence (an incidence of 60% to 92%), mostly occurs when patients are waiting for elective surgery [4]. Preoperative anxiety has been identified as the critical factor associated with abnormality of hemodynamics and the sympathetic system, parasympathetic system, and endocrine system [5–8]. In addition, a high level of preoperative anxiety has been reported as one of the risk factors for prognosis [9, 10] resulting in postoperative nausea and vomiting (PONV) and patient dissatisfaction [11, 12].

Notably, existing surveys have documented that preoperative anxiety is related to age, sex, surgical procedures, pain level, ASA (American Society of Anesthesiologists) grade, depressive symptoms, psychiatric disorders, and cancer history [1, 13]. In general, those factors related to preoperative anxiety can be classified into the environmental (e.g., pain) and genetic factors (e.g., race and sex). Understanding risk factors triggering preoperative anxiety could benefit clinicians in effective treatment and intervention of anxiety. Furthermore, a variety of factors interrelating and inducing the occurrence of preoperative anxiety must be known and considered into the methodology of coping with preoperative anxiety. From the aspect of psychiatric genetics, published studies have revealed gene-environment interaction is the determinant of anxiety and anxiety-relevant disorders [14, 15]. ABO blood types are inherited from parents and ancestors, which to some extent illustrate genetic characteristics. There is a current paucity of observational studies investigating the relationship between preoperative anxiety and phenotypic factors such as ABO blood types.

ABO blood types are composed of antigenic, polymorphic, and genetic substances which are found on the surface of Red Blood Cells (RBCs) as well as some other tissues and cells [16, 17]. Previous investigations have found the ABO blood types correlate with many clinical conditions including diabetes, diverse cancer, and cardiovascular diseases [18–21], which indicate the underlying correlations of ABO blood types with some diseases. Based on the above narrations, we assumed the connection between ABO blood types and preoperative anxiety. Currently, few studies have explored the association between the ABO blood types and preoperative anxiety.

Therefore, in the present study, we identified whether ABO phenotype influences the preoperative anxiety level in patients undergoing elective surgery. Hospital anxiety and depression scale (HADS) [22] and visual analogue scales for anxiety (VA) [23], as validated tools, were used to examine the preoperative anxiety level. Theoretically, this work was conducted to detect the effect of diverse ABO phenotypes on preoperative anxiety.

## 2. Materials and Methods

### 2.1. Study Design and Ethics Authorization

This cross-sectional study was conducted in the First Affiliated Hospital, School of Medicine, Shihezi University, between December 2018 and June 2019. The procedure of the study was granted by the Ethics Committee of the First Affiliated Hospital, School of Medicine, Shihezi University. This study was performed in accordance with the Helsinki Declaration, as revised in 1989. This observational trial enrolled anonymized patients and had no private information to identify people. This study protocol was also approved by the Chinese Clinical Trial Registry (ChiCTR1800019390).

### 2.2. Eligibility Criteria of Participants

The inclusion criteria are as follows: patients scheduled for elective surgery or noncardiac surgery, patients aged 18–65 years, patients of Han Chinese race, patients with ASA (American Society of Anesthesiologists) grades I–III, and patients scheduled for general anesthesia.

The exclusion criteria are as follows: patients with deficit of cognition, reading, writing, or language; patients with menstruation, suffering from climacterium, or who are pregnant; patients administered with sedative, antidepressants, or analgesics; and patients who cannot cooperate with the data collector or suffering from psychiatric illness and metal disorders.

### 2.3. Sample Size Calculation

This study included 352 patients. The numbers of the A, B, AB, and O blood type groups were 93, 86, 88, and 85, respectively. For the sake of the proof of enough sample size, we have used PASS software to calculate the effect power. The means and SD (standard deviation) were all obtained from 352 patients. The means of the A, B, AB, and O blood type groups were 5.81, 4.69, 4.56, and 4.98, respectively. The SDs of the A, B, AB, and O blood type groups were 1.70, 1.81, 1.94, and 1.93, separately. The significance level (*α*) was also set at 0.05. In the one-way ANOVA study in PASS software, sample sizes of 85, 85, 85, and 85 were set in the A, B, AB, and O blood type groups, respectively. The total sample of 340 subjects in the four groups can achieve a power of 98% to detect the statistical difference of preoperative anxiety level among the four groups.

### 2.4. Outcome Measures

In this study, the major outcome was preoperative anxiety or stress level including VA scores and HADS scores in the A, B, AB, and O blood type groups.

The secondary outcome was the correlation between ABO blood types and VA scores or HADS scores.

### 2.5. Questionnaire and Scores

VA scores and HADS scores [22, 24, 25], a reliable strategy to estimate preoperative anxiety, were used to assess preoperative anxiety. VA is in a scale from 0 (no anxiety) to 10 (worst anxiety), in which higher scores indicate being more anxious. In detail, a score of 0 to 4 means normal or not and a score of 5 to 10 means moderate or severe preoperative anxiety.

In HADS scores, 14 items are averagely classified into 2 subscales including HADS-A (anxiety) and HADS-D (depression). The items of HADS-A are worry, tension, panic, fear, restlessness, and difficulties in relaxing. The items of HADS-D mainly assess anhedonia (not experiencing joy). For each item, the scores range from 0 to 3, with higher scores suggesting higher severity. In detail, a score of 0 to 7 means normal or not, a score of 8 to 10 means mild, a score of 11 to 14 means moderate, and a score of 15 to 21 means severe [26].

ASA grade is the strategy of estimating the anesthetic risk before surgery and ranges from I to VI [27, 28]. The higher ASA grade means higher severity in physical status and anesthetic risk.

VAS (visual analogue scales for pain) is the predictive tool to assess the pain level [29]. VAS is in the scale from 0 (no pain) to 10 (worst pain), in which higher scores indicate more pain [30].

### 2.6. Data Acquisition

The online questionnaire was used to record clinical information via phones and tablet computer. Before surgery, age, race, sex, ASA grade, BMI (body mass index), operation type, operation history, level of pain (VAS), anesthetic strategy, ABO blood types, and clinical history of patients were recorded online by anesthetists. When entering the operative room, patients filled in the VA scores and HADS scores, in forenoon.

### 2.7. Data Analysis

Data analysis was conducted by using SPSS version 22.0. Descriptive analysis was performed to identify the number and percentage of demographic characteristics. A one-way ANOVA, chi-square test, or Kruskal-Wallis pairwise comparisons were used to identify the difference among the A, B, AB, and O blood type groups in some variables. Bivariate correlation analysis (Spearman's correlation and Pearson's correlation) was harnessed to estimate the interrelation between some variables (e.g., ABO blood types) and preoperative anxiety (VA scores and HADS-A scores). The independent variables with *P* < 0.1 in bivariate correlation analysis were enrolled in binary logistic regression for further analysis. A logistic regression model was used to examine the effect of some independent variables on preoperative anxiety level. The method of backward LR was employed in binary logistic regression. In a binary logistic regression model, VA scores ranging from 1 to 4 were set to zero, with 169 patients. VA scores ranging from 5 to 10 were set to one, with 183 patients. Ultimately, five variables including age, sex, ASA, VAS, and ABO blood types were included in the binary logistic regression model. With adequate sample volume, there is no overfitting relation in the binary logistic regression model of this survey. The odds ratio and 95% confidence interval (CI) were used to assess the power of association. The statistical difference in different groups and the significant correlation in variables were set at *P* < 0.05.

## 3. Results

### 3.1. Patient Demographics

Age, sex, BMI, ASA grade, VAS, operation history, operation type, ABO blood types, smoking history, hypertension, and diabetes were all recorded as the baseline before surgery. The number and percentage of these items were used to describe and measure the patient demographics in the A, B, AB, and O blood type groups, as shown in Table 1. Four groups displayed no significant difference in demographic characters and clinical information.

### 3.2. VA Score Measurement in the A, B, AB, and O Blood Type Groups before Surgery

**Figure 1:**
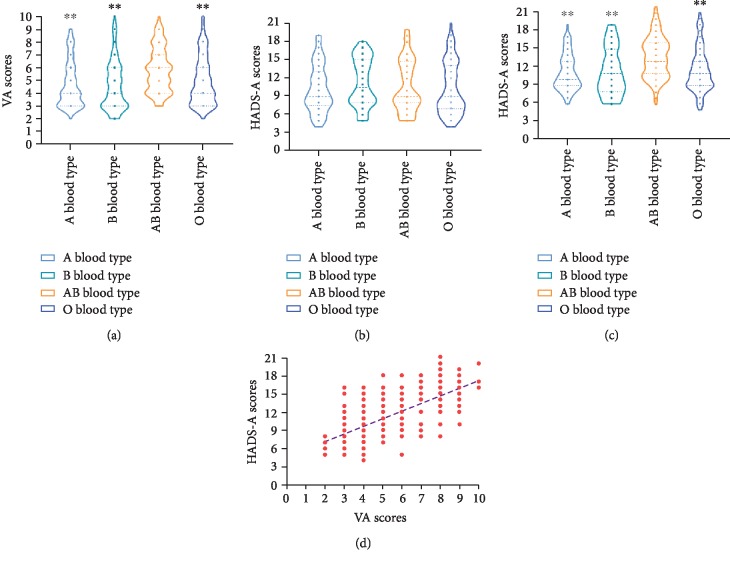
VA (a), HADS-A (b), and HADS-D (c) score measurement in the A, B, AB, and O blood type groups before surgery and bivariate correlation analysis between preoperative VA scores and HADS-A scores (d). Significant difference, ^∗^*P* < 0.05 and ^∗∗^*P* < 0.01 vs. the AB group.

VA scores were used to assess preoperative anxiety in the A, B, AB, and O blood type groups. Then, we analyzed the effect of different blood types on VA scores. When compared with the A, B, and O blood type groups, the AB blood type group has the higher VA scores (median [25th, 75th] = 6 [4, 7] of the AB group vs. 4 [3, 6] of the A group, *P* < 0.01; 4 [3, 6] of the B group, *P* < 0.01; and 4 [3, 6] of the O group, *P* < 0.01), as shown in Figure 1(a).

### 3.3. HADS-A Score Measurement in the A, B, AB, and O Blood Type Groups before Surgery

To further identify the relationship between ABO blood types and preoperative anxiety, HADS-A scores were also employed to estimate the level of anxiety. In comparison to the A, B, and O blood type groups, the AB blood type group has the higher HADS-A scores (12 [10, 15] of the AB group vs. 9 [8, 12] of the A group, *P* < 0.01; 10 [7, 14] of the B group, *P* < 0.01; and 10 [8, 12] of the O group, *P* < 0.01), as shown in Figure 1(b).

### 3.4. HADS-D Score Measurement in the A, B, AB, and O Blood Type Groups before Surgery

To identify the relationship between ABO blood types and preoperative depression, HADS-D scores were employed to estimate the level of depression. One-way ANOVA analysis showed no apparent difference of HADS-D scores in the four groups (9 [8, 14] of the AB group, 9 [7.5, 13] of the A group, 10.5 [8, 15] of the B group, and 9 [7, 14] of the O group, with *P* > 0.05), as shown in Figure 1(c).

### 3.5. Bivariate Correlation Analysis of Factors Correlated with Preoperative VA Scores

To examine ABO blood types related to preoperative anxiety, bivariate correlation analysis was employed to assess the relationship between preoperative VA scores and ABO blood types or other variables. In bivariate correlation analysis, age, sex, BMI, ASA grade, VAS, operation history, operation type, ABO blood types, smoking history, hypertension, and diabetes were included. Ultimately, the independent variables with *P* < 0.1 were age (correlation coefficient: -0.11, *P* < 0.05), sex (correlation coefficient: 0.29, *P* < 0.01), ASA grade (correlation coefficient: -0.14, *P* < 0.05), VAS (correlation coefficient: 0.19, *P* < 0.01), and ABO blood types (correlation coefficient: 0.13, *P* < 0.05), which were correlated with preoperative VA scores (Table 2).

### 3.6. Bivariate Correlation Analysis of Factors Correlated with Preoperative HADS-A Scores

To further examine ABO blood types related to preoperative anxiety, bivariate correlation analysis was also used to estimate the relationship between preoperative HADS-A scores and ABO blood types or other variables. In bivariate correlation analysis, age, sex, BMI, ASA grade, VAS, operation history, operation type, ABO blood types, smoking history, hypertension, and diabetes were also included. Finally, the independent variables with *P* < 0.1 were age (correlation coefficient: -0.13, *P* < 0.05), sex (correlation coefficient: 0.18, *P* < 0.01), ASA grade (correlation coefficient: -0.11, *P* < 0.05), VAS (correlation coefficient: 0.15, *P* < 0.01), and ABO blood types (correlation coefficient: 0.11, *P* < 0.05), which were correlated with preoperative HADS-A scores (Table 2).

### 3.7. Bivariate Correlation Analysis between Preoperative VA Scores and HADS-A Scores

After bivariate correlation analysis, both VA scores and HADS-A scores were associated with age, sex, ASA grade, VAS, and ABO blood types. Thus, in our study, we assumed that VA scores and HADS-A scores displayed consistency in estimating the preoperative anxiety level. In bivariate correlation analysis, VA scores are linearly correlated with HADS-A scores (correlation coefficient: 0.67, *P* < 0.01). Linear fitting equation is *y* = 1.24*x* + 4.65, as shown in Figure 1(d).

### 3.8. Logistic Regression Model to Examine the Effect of Variables on Preoperative Anxiety Level

Considering that VA scores are linearly correlated with HADS-A scores, only the VA score was used in the logistic regression model to determine the effect of independent variables on the preoperative anxiety level. After bivariate correlation analysis, we confirmed that the independent variables which were related to preoperative VA scores were age, sex, ASA grade, VAS, and ABO blood types. First, based on the criteria of VA scores, we converted measurement data into dichotomous data. In detail, patients with VA scores lower than 5 were regarded as having no preoperative anxiety. Patients with VA scores greater than 4 were regarded as having preoperative anxiety.

In the backward binary logistic regression analysis, the model with sex, ASA, VAS, and ABO blood types was apparently correlated with VA scores. The Hosmer and Lemeshow test, with a *P* value of 0.70, effectively indicated the better model goodness of fit. The ratio of having preoperative anxiety was 3.73 (95% CI: 2.32-6.00, *P* < 0.001) times in female patients than in male patients; 0.36 (95% CI: 0.21-0.63, *P* < 0.001) times in patients with ASA grade II than in patients with ASA grade I; 0.41 (95% CI: 0.20-0.86, *P* < 0.05) times in patients with ASA grade III than in patients with ASA grade I; 1.25 (95% CI: 1.11-1.41, *P* < 0.001) times in patients with higher VAS scores than in patients with lower VAS scores; and 0.28 (95% CI: 0.16-0.49, *P* < 0.01) times in patients with non-AB blood type than in patients with AB blood type (Table 3).

## 4. Discussion

At present, few investigations depicted the potential relationship between ABO blood types and preoperative anxiety, after literature retrieval. This observational study was the first one to report the relationship between ABO blood type groups and preoperative anxiety quantified by VA scores and HADS-A scores. With enough sample size of 352 patients, our work provided strong evidence that ABO blood type groups are associated with preoperative anxiety, from a variety of statistical methods. The major results of this study are as follows. First, the difference in ABO blood type correlates with the preoperative anxiety level. Specifically, AB blood type patients undergoing elective surgery are susceptible to preoperative anxiety and AB blood type is the risk factor for preoperative anxiety. Second, in our study, VA scores are linearly interrelated with HADS-A scores. Third, sex, ASA grade, VAS, and ABO blood types are identified as the factors which were correlated with preoperative anxiety.

ABO blood types, as the indirective indicator of genetics, were identified to have a critical role in mediating numerous medical conditions, including tumors, immune diseases, cardiovascular diseases, endocrine diseases, infectious diseases, and mental disorders [31–37]. From aspects of affective disorder, previous studies of psychiatric diagnoses found that blood group O was significantly associated with involutional melancholia [38]. In the study of 108 normal volunteers, the results indicated the correlation between O blood type and depression [39]. A current trial of 8842 pregnant women reported that, in comparison to those with blood type B, patients with blood type O, A, or AB had a higher odds ratio of postpartum depression [36]. Additionally, past surveys established that mental anxiety with cardiovascular risk has also associated with ABO blood type [40]. Hence, ABO blood types play the essential role in affective disorders. Similarly, in our study, we also proved the effect of ABO blood type on affective disorders (preoperative anxiety) from multiple aspects. When exposed to a preoperative stressor, patients with blood group AB displayed their vulnerability and fragility. After summarizing the above evidence, different ABO phenotypes inherit the genetic characteristics in affective disorder from the ancestors and display specific performances.

In the realm of psychiatric disorder, numerous studies have reported that anxiety disorder is heritable and familial to some extent. Currently, the gene-environment interaction is identified as the determinant of anxiety and anxiety disorders [15, 41, 42]. DNA variations including single-nucleotide variants (SNVs or SNPs) are widely used in the association analysis of psychiatric genetics [43, 44]. Genome-wide association studies (GWAS) of military veterans illustrated the genome-wide significant association between SNPs of rs8042149 in the RORA (retinoid-related orphan receptor gene) and PTSD (posttraumatic anxiety disorder) [45]. In an imaging genetic study, some genes were related with the amygdala activation elicited by anxiety or anxiety-related stimulation, such as SLC6A4 (solute carrier family 6 member 4), BDNF (brain-derived neurotrophic factor) val66met, MAOA (monoamine oxidase A), and COMT (catechol-O-methyltransferase) val158met [46]. Different ABO blood types are considered the genetic polymorphism among individuals, to some degree. According to the theory of the gene-environment interaction, in our study, we provided the evidence that ABO blood groups correlate with preoperative anxiety and the AB blood group is the functional “gene variant” associated with high reactivity in preoperative anxiety. Notably, ABO blood group antigens are catalyzed via glycosyltransferases including GTA (alpha 1-3-N-acetylgalactosaminyltransferase) and GTB (alpha 1-3-galactosyltransferase) [47]. Thus, different ABO blood types show different activities of glycosyltransferases. Glycosyltransferases were reported in significant repair and regeneration in nerve injury, via glycosylation of glycoproteins in Schwann cells [48, 49]. Moreover, glycosyltransferases are also involved in neuroinflammatory response [50]. Meanwhile, neuroinflammatory plays the critical role in inducing the anxiety disorder [51]. From the aspect of neuroinflammatory and nerve myelination, it can be hypothesized that the effect of glycosyltransferases on the synthesis of antigens of ABO blood groups contributes to various preoperative anxiety patterns. In addition, the difference of blood type in preoperative hormone levels and pain in a certain blood group [52, 53] could indicate plausible and underlying physiopathologic reasons for the correlation between ABO blood type and preoperative anxiety.

Merits of this study are described as follows. First, both VA scores and HADS scores were utilized to examine the preoperative anxiety level, which can effectively reduce measurement bias in preoperative anxiety assessment. Second, before binary logistic regression analysis of independent variables related to preoperative anxiety, bivariate correlation analysis was harnessed to eliminate unrelated variables and increase the accuracy and efficiency of logistic regression analysis. Third, in logistic regression analysis and bivariate correlation analysis, ABO blood types were all proved to apparent association with preoperative anxiety level, which offers strong evidence for our primary rationale. MCID (minimal clinically important difference), as the indispensable index, along with a *P* value, is used to describe the clinical significance of differences in studies. According to a previous study, there was a 1.5 MCID [54]. MCID values between the AB blood type group and other groups were all greater than 1.5, which strongly suggest that this study has the value of guiding the management of perioperative anxiety.

However, this study also has some limitations. Firstly, blood types are mainly classified into the A1 and A2 groups. This investigation failed to explore the relationship between subtypes of ABO blood type with preoperative anxiety. Moreover, Rh (Rhesus) blood types are also another indispensable blood-group system in transfusion. Our work still did not describe the potential role of Rh blood type in preoperative anxiety. In this study, we merely record 3 patients with Rh-negative blood type. In a word, in a future work, a large sample size should be supplemented to identify the role of ABO blood type subtype and Rh blood types in preoperative anxiety response and depression disorder.

## 5. Conclusions

Differences in ABO blood types were found in preoperative anxiety (VA scores and HADS scores), and the AB group displayed a high preoperative anxiety level. ABO blood types, sex, ASA grade, and VAS were associated with preoperative anxiety in patients undergoing elective surgery.

## Figures and Tables

**Table 1:** Demographic characteristics of patients.

Variables	Response	A (*n* = 93)	B (*n* = 86)	AB (*n* = 88)	O (*n* = 85)
Age, yr (mean ± SD)		42.12 ± 9.04	44.06 ± 10.22	42.17 ± 9.56	42.65 ± 8.93

Sex	Male	47 (51%)	41 (48%)	44 (50%)	41 (48%)
	Female	46 (49%)	45 (52%)	44 (50%)	44 (52%)

BMI (mean ± SD)		24.42 ± 3.34	24.65 ± 4.01	24.77 ± 3.64	25.28 ± 3.48

ASA (%)	I	27 (29%)	23 (27%)	26 (30%)	27 (32%)
	II	52 (56%)	48 (56%)	46 (52%)	45 (53%)
	III	14 (15%)	15 (17%)	16 (18%)	13 (15%)

Operation history (%)	0	61 (66%)	58 (67%)	55 (63%)	58 (68%)
	1	26 (28%)	23 (27%)	26 (29%)	21 (25%)
	≥2	6 (6%)	5 (6%)	7 (8%)	6 (7%)

Smoking (%)	Yes	18 (19%)	17 (20%)	19 (22%)	15 (18%)
	No	75 (81%)	69 (80%)	69 (78%)	70 (82%)

VAS (%)	1~3	47 (51%)	46 (53%)	49 (56%)	47 (55%)
	≥4	46 (49%)	40 (47%)	39 (44%)	38 (45%)

Operation type (%)	Liver	20 (21%)	21 (24%)	18 (20%)	17 (20%)
	Gallbladder	13 (14%)	11 (14%)	14 (16%)	9 (11%)
	Intestines	60 (65%)	54 (63%)	56 (64%)	59 (69%)

Hypertension (%)	Yes	11 (12%)	15 (17%)	16 (18%)	14 (16%)
	No	82 (88%)	71 (83%)	72 (82%)	71 (84%)

Diabetes (%)	Yes	6 (6%)	3 (3%)	5 (6%)	6 (7%)
	No	87 (94%)	83 (97%)	83 (94%)	79 (93%)

Note: one-way ANOVA and chi-square test were used to identify the difference among the A, B, AB, and O blood type groups in some variables. BMI = body mass index; ASA = American Society of Anesthesiologists; VAS = visual analogue scale for pain; SD = standard deviation; yr = year.

**Table 2:** Bivariate analysis of factors correlated with VA scores and HADS-A scores.

Independent variables	Dependent variables			
	VA scores		HADS-A scores	
	Correlation coefficient	*P* value	Correlation coefficient	*P* value
Age	-0.11	0.034^∗^	-0.13	0.019^∗^
Sex	0.29	<0.001^∗^	0.18	<0.001^∗^
BMI	0.020	0.70	0.01	0.86
ASA	-0.14	0.011^∗^	-0.11	0.035^∗^
Operation history	0.033	0.53	0.052	0.33
Smoking	0.015	0.78	0.0091	0.87
VAS	0.19	<0.001^∗^	0.15	0.006^∗^
Operation type	0.002	0.97	0.027	0.62
Hypertension	0.024	0.65	-0.0050	0.93
Diabetes	0.066	0.22	0.041	0.44
Blood types	0.13	0.012^∗^	0.11	0.032^∗^

Note: Spearman's correlation analysis and Pearson's correlation analysis used in bivariate analysis. Significant difference between independent variables and dependent variables, ^∗^*P* < 0.1 (*n* = 352). BMI = body mass index; ASA = American Society of Anesthesiologists; VAS = visual analogue scale for pain.

**Table 3:** Binary logistic regression analysis of factors correlated with VA scores.

Variables	Response	Odds ratio (95% CI)	*P* value
Age		1.00 (0.97-1.02)	0.71

Sex	Male	1	
	Female	3.73 (2.32-6.00)	<0.001^∗^

ASA	I	1	
	II	0.36 (0.21-0.63)	<0.001^∗^
	III	0.41 (0.20-0.86)	0.019^∗^

VAS		1.25 (1.11-1.41)	<0.001^∗^

Blood types	AB	1	
	A/B/O	0.28 (0.16-0.49)	0.001^∗^

Note: backward LR was used in binary logistic regression analysis of factors correlated with VA scores. In the binary logistic regression model, VA scores ranging from 1 to 4 were set at zero, with 169 patients. VA scores ranging from 5 to 10 were set at one, with 183 patients. Significant difference, ^∗^*P* < 0.05 (*n* = 352). ASA = American Society of Anesthesiologists; VAS = visual analogue scale for pain.

## Data Availability

No additional data are available.

## References

[1] Badner N. H., Nielson W. R., Munk S., Kwiatkowska C., Gelb A. W. (1990). Preoperative anxiety: detection and contributing factors. *Canadian Journal of Anaesthesia*.

[2] Jawaid M., Mushtaq A., Mukhtar S., Khan Z. (2007). Preoperative anxiety before elective surgery. *Neurosciences*.

[3] Mulugeta H., Ayana M., Sintayehu M., Dessie G., Zewdu T. (2018). Preoperative anxiety and associated factors among adult surgical patients in Debre Markos and Felege Hiwot referral hospitals, Northwest Ethiopia. *BMC Anesthesiology*.

[4] Anna P., Sucharita C., Pirjo M. (2009). Preoperative anxiety in neurosurgical patients. *Journal of Neurosurgical Anesthesiology*.

[5] Takagi H., Ando T., Umemoto T., ALICE (All-Literature Investigation of Cardiovascular Evidence) Group (2017). Perioperative depression or anxiety and postoperative mortality in cardiac surgery: a systematic review and meta-analysis. *Heart and Vessels*.

[6] Oyola M. G., Handa R. J. (2017). Hypothalamic-pituitary-adrenal and hypothalamic-pituitary-gonadal axes: sex differences in regulation of stress responsivity. *Stress*.

[7] Sleigh J. W., Henderson J. D. (2010). Heart rate variability and preoperative anxiety. *Acta Anaesthesiologica Scandinavica*.

[8] Sevenster D., Hamm A., Beckers T., Kindt M. (2015). Heart rate pattern and resting heart rate variability mediate individual differences in contextual anxiety and conditioned responses. *International Journal of Psychophysiology*.

[9] Tully P. J., Bennetts J. S., Baker R. A., McGavigan A., Turnbull D. A., Winefield H. R. (2011). Anxiety, depression, and stress as risk factors for atrial fibrillation after cardiac surgery. *Heart & Lung*.

[10] Li S., Qi M., Yuan W., Chen H. (2015). The impact of the depression and anxiety on prognosis of cervical total disc replacement. *Spine*.

[11] Ali A., Lindstrand A., Sundberg M., Flivik G. (2017). Preoperative anxiety and depression correlate with dissatisfaction after total knee arthroplasty: a prospective longitudinal cohort study of 186 patients, with 4-year follow-up. *The Journal of Arthroplasty*.

[12] Bosch J. E., Den V., Moons K. G., Bonsel G. J., Kalkman C. J. (2005). Does measurement of preoperative anxiety have added value for predicting postoperative nausea and vomiting?. *Anesthesia & Analgesia*.

[13] Caumo W., Schmidt A. P., Schneider C. N. (2010). Risk factors for preoperative anxiety in adults. *Acta Anaesthesiologica Scandinavica*.

[14] Nugent N. R., Tyrka A. R., Carpenter L. L., Price L. H. (2011). Gene-environment interactions: early life stress and risk for depressive and anxiety disorders. *Psychopharmacology*.

[15] Sumeet S., Abigail P., Bekh B., Ressler K. J. (2016). Gene × environment determinants of stress- and anxiety-related disorders. *Annual Review of Psychology*.

[16] Meo S. A., Suraya F., Jamil B. (2017). Association of ABO and Rh blood groups with breast cancer. *Saudi Journal of Biological Sciences*.

[17] Meo S. A., Rouq F. A., Suraya F., Zaidi S. Z. (2016). Association of ABO and Rh blood groups with type 2 diabetes mellitus. *European Review for Medical and Pharmacological Sciences*.

[18] Franchini M., Liumbruno G. M., Lippi G. (2016). The prognostic value of ABO blood group in cancer patients. *Blood Transfusion*.

[19] Stakišaitis D., Juknevičienė M., Ulys A. (2018). ABO blood group polymorphism has an impact on prostate, kidney and bladder cancer in association with longevity. *Oncology Letters*.

[20] Zhang H., Mooney C. J., Reilly M. P. (2012). ABO blood groups and cardiovascular diseases. *International Journal of Vascular Medicine*.

[21] Qi L., Cornelis M. P., Jensen M. (2010). Genetic variants in ABO blood group region, plasma soluble E-selectin levels and risk of type 2 diabetes. *Human Molecular Genetics*.

[22] Hernández-Palazón J., Fuentes-García D., Falcón-Araña L. (2017). Assessment of Preoperative Anxiety in Cardiac Surgery Patients Lacking a History of Anxiety: Contributing Factors and Postoperative Morbidity. *Journal of Cardiothoracic and Vascular Anesthesia*.

[23] Delewi R., Rohling W. J., Wagenaar T. C. (2016). Anxiety levels of patients undergoing coronary procedures in the catheterization laboratory. *International Journal of Cardiology*.

[24] Facco E., Stellini E., Bacci C. (2013). Validation of visual analogue scale for anxiety (VAS-A) in preanesthesia evaluation. *Minerva Anestesiologica*.

[25] Beekman E., Verhagen A. (2018). Clinimetrics: hospital anxiety and depression scale. *Journal of Physiotherapy*.

[26] Whelan-Goodinson R., Ponsford J., Schönberger M. (2009). Validity of the hospital anxiety and depression scale to assess depression and anxiety following traumatic brain injury as compared with the structured clinical interview for DSM-IV. *Journal of Affective Disorders*.

[27] Anastasios K., Heather G., Smith A. B., Mannion C., Ong T. K., Mitchell D. (2010). ASA grade and disease-free mortality in head and neck cancer patients: a prospective study. *British Journal of Oral & Maxillofacial Surgery*.

[28] Guo R., Yu W., Meng Y. (2016). Correlation of ASA grade and the Charlson comorbidity index with complications in patients after transurethral resection of prostate. *Urology*.

[29] Hirschfeld G., Zernikow B. (2013). Cut points for mild, moderate, and severe pain on the VAS for children and adolescents: what can be learned from 10 million ANOVAs?. *Pain*.

[30] O'Connell Ferster A. P., Hu A. (2018). Perceptions of pain of laryngeal electromyography. *The Laryngoscope*.

[31] Dubinski D., Won S.-Y., Behmanesh B. (2018). Influence of ABO blood type on the outcome after non-aneurysmal subarachnoid hemorrhage. *Acta Neurochirurgica*.

[32] ZGK İ., Unal M. (2018). Is there an association of ABO blood groups and Rhesus factor with alopecia areata?. *Journal of Cosmetic Dermatology*.

[33] Park S., Kim K. S., Kim J. S. (2017). Prognostic value of ABO blood types in young patients with breast cancer; a nationwide study in Korean Breast Cancer Society. *Medical Oncology*.

[34] Paquette M., Dufour R., Baass A. (2017). ABO blood group is a cardiovascular risk factor in patients with familial hypercholesterolemia. *Journal of Clinical Lipidology*.

[35] Fagherazzi G., Gusto G., Clavel-Chapelon F., Balkau B., Bonnet F. (2015). ABO and Rhesus blood groups and risk of type 2 diabetes: evidence from the large E3N cohort study. *Diabetologia*.

[36] Song C., Leng J., Wang L. (2018). ABO blood types and postpartum depression among Chinese women: a prospective cohort study in Tianjin, China. *Women & Health*.

[37] Anstee D. J. (2010). The relationship between blood groups and disease. *Blood*.

[38] Irvine D. G., Miyashita H. (1965). Blood types in relation to depressions and schizophrenia: a preliminary report. *Canadian Medical Association Journal*.

[39] Singg S., Lewis J. L. (2001). Depression and blood types. *Psychological Reports*.

[40] Neumann J. K., Arbogast B. W., Chi D. S., Arbogast L. Y. (1992). Effects of stress and blood type on cortisol and VLDL toxicity preventing activity. *Psychosomatic Medicine*.

[41] Shimada-Sugimoto M., Otowa T., Hettema J. M. (2015). Genetics of anxiety disorders: genetic epidemiological and molecular studies in humans. *Psychiatry and Clinical Neurosciences*.

[42] Klengel T., Pape J., Binder E. B., Mehta D. (2014). The role of DNA methylation in stress-related psychiatric disorders. *Neuropharmacology*.

[43] Smoller J. W. (2015). The genetics of stress-related disorders: PTSD, depression, and anxiety disorders. *Neuropsychopharmacology*.

[44] Demirkan A., Penninx B. W., Hek K. (2011). Genetic risk profiles for depression and anxiety in adult and elderly cohorts. *Molecular Psychiatry*.

[45] Logue M. W., Baldwin C., Guffanti G. (2013). A genome-wide association study of post-traumatic stress disorder identifies the retinoid-related orphan receptor alpha (*RORA*) gene as a significant risk locus. *Molecular Psychiatry*.

[46] Domschke K., Dannlowski U. (2010). Imaging genetics of anxiety disorders. *NeuroImage*.

[47] Alfaro J. A., Zheng R. B., Persson M. (2008). ABO(H) blood group A and B glycosyltransferases recognize substrate via specific conformational changes. *Journal of Biological Chemistry*.

[48] Yang H., Yan M., Cheng C. (2010). Expression of *β*-1,4-galactosyltransferase I in rat Schwann cells. *The Journal of Biological Chemistry*.

[49] Aiguo S., Jun Y., Fei D., Gu X., Zhu D., Gu J. (2003). Overexpression of *β*-1,4-galactosyltransferase I in rat Schwann cells promotes the growth of co-cultured dorsal root ganglia. *Neuroscience Letters*.

[50] Meijuan Y., Chunlin X., Shuqiong N. (2007). The role of TNF-*α* and its receptors in the production of *β*-1,4 galactosyltransferase I and V mRNAs by rat primary astrocytes. *Journal of Molecular Neuroscience*.

[51] Furtado M., Katzman M. A. (2015). Neuroinflammatory pathways in anxiety, posttraumatic stress, and obsessive compulsive disorders. *Psychiatry Research*.

[52] Simoni A. H., Jerwiarz A., Randers A., Gazerani P. (2018). Association between ABO blood types and pain perception. *Somatosensory & Motor Research*.

[53] Reimold M., Knobel A., Rapp M. A. (2011). Central serotonin transporter levels are associated with stress hormone response and anxiety. *Psychopharmacology*.

[54] Carson S. S., Cox C. E., Wallenstein S. (2016). Effect of palliative care–led meetings for families of patients with chronic critical illness: a randomized clinical trial. *Journal of the American Medical Association*.

